# Urban Filter vs. Natural Refuge: Divergent Diptera Community Assembly Mechanisms—Evidence from Beijing, China

**DOI:** 10.3390/biology15110865

**Published:** 2026-05-30

**Authors:** Boyu Fang, Zihao Zhang, Yuwei Ding, Jiaxuan Cheng, Jun Yang, Jingyu Zhai, Xiaole Chen, Ayman Khamis Elsayed, Makoto Tokuda, Ding Yang, Yunhui Liu, Rudolf Meier, Qinggang Wang, Xuankun Li

**Affiliations:** 1 State Key Laboratory of Agricultural and Forestry Biosecurity, College of Plant Protection, China Agricultural University, Beijing 100193, China; q1605613231q@gmail.com (B.F.); ultrainsect@163.com (Z.Z.); yuweiding2025@163.com (Y.D.); dyangcau@126.com (D.Y.); 2 Department of Ecological Science and Engineering, College of Resources and Environmental Sciences, China Agricultural University, Beijing 100193, China; cjx28282828@163.com (J.C.); liuyh@cau.edu.cn (Y.L.); 3 Department of Resource Management, Capital Green Culture Stele Forest Management Office, Beijing 100193, China; junyang45677@163.com; 4 Department of Horticulture and Technology, Management Office of Zizhuyuan Park, Beijing 100193, China; m15510055610_1@163.com (J.Z.); chenxiaole2016@163.com (X.C.); 5 Laboratory of Systems Ecology, Faculty of Agriculture, Saga University, Saga 840-8502, Japan; ayman.khamis77@gmail.com (A.K.E.); tokudam@cc.saga-u.ac.jp (M.T.); 6 The United Graduate School of Agricultural Sciences, Kagoshima University, Kagoshima 890-0065, Japan; 7 Center for Integrative Biodiversity Discovery, Leibniz-Institut für Evolutions-und Biodiversitätsforschung, Museum für Naturkunde, 10115 Berlin, Germany; rudolf.meier@mfn.berlin; 8 Institute for Biology, Humboldt University Berlin, 10115 Berlin, Germany

**Keywords:** DNA barcoding, environmental filtering, β-diversity partitioning, trophic guilds, urban biodiversity, community assembly

## Abstract

Urbanization can act as a powerful ecological filter, restructuring biodiversity through species loss, replacement, and altered resource pathways. While urban green spaces (UGS) are recognized as potential biodiversity refuges, the effectiveness and mechanisms for conserving insect diversity across the urban-to-natural gradient remain poorly understood. Here, we combine full-season Malaise trapping (April–November) with MinION-based DNA barcoding to test two predictions about how urbanization reshapes Diptera communities across five sites in Haidian District, Beijing, ranging from residential areas and urban parks to a nearby shallow mountain reserve (BWM). Based on 5528 barcoded individuals, we identified 686 putative species from 39 families. As predicted, β-diversity between urban and mountain sites was overwhelmingly driven by species turnover rather than nestedness, demonstrating that cities do not simply receive subsets of the surrounding fauna but actively reassemble communities. This filtering effect was, however, trophic-guild specific. Detritivores showed the highest replacement, consistent with a shift from natural to anthropogenic resource subsidies, while predators/parasitoids exhibited significant nested loss, aligning with their hypothesized sensitivity at higher trophic levels. Vegetation structure further clarified these patterns: vegetation density, not plant species richness, was the primary bottom-up driver for herbivore and predator/parasitoid diversity, whereas detritivores were decoupled from living plant biomass. These findings demonstrate that urban and near-natural habitats maintain distinct species pools via guild-specific assembly pathways, highlighting the need for guild-specific conservation strategies for urban biodiversity conservation. Extending beyond compositional analysis, we propose a temporal-abundance framework, classifying species by persistence and abundance, as a diagnostic tool for assessing ecological integrity and guiding conservation in urbanizing landscapes.

## Simple Summary

As cities expand, urban green spaces are increasingly relied upon to protect wildlife. However, it remains unclear if these areas truly support the same insect communities as natural habitats. We studied flies and midges—a highly diverse but often overlooked group—across urban parks and a nearby natural mountain in Beijing. Using advanced DNA sequencing, we tracked over 600 species from spring to autumn. We found that urban environments do not simply have fewer natural species; instead, they host entirely different insect communities. Interestingly, how the city affects these insects depends heavily on their diet. Decomposers (insects feeding on dead organic matter) in the city are replaced by new, urban-adapted species. In contrast, predatory and parasitic insects are highly vulnerable and simply vanish from urban areas. Furthermore, city environments lack the natural “seasonal bursts” of diverse insects found in the mountains, relying instead on a few dominant, year-round species. Our findings show that effective urban conservation cannot be a one-size-fits-all approach. We must tailor our efforts—such as retaining leaf litter for decomposers and maintaining diverse tree canopies to save predators—to ensure healthier and more balanced urban ecosystems.

## 1. Introduction

Over the past few decades, rapid global urbanization has profoundly transformed landscapes [1,2], creating a stark ecological contrast between densely built urban areas and adjacent natural habitats [3,4,5]. This transformation drives fundamental changes in biodiversity, making the relationship between urban and nearby natural communities a central question in urban ecology [6,7,8,9]. Urban green spaces (UGS), such as parks, gardens, forest remnants, and roadside vegetation, are widely recognized as potential refuges and corridors for biodiversity within cities [10,11,12]. Given the dispersal capacity of many insects, the composition of urban communities is often considered to be strongly influenced by their distance from nearby natural habitats [13]. Beyond spatial configuration, local features such as plant species richness and vegetation cover are also thought to play a key role in shaping insect communities [14,15]. Yet, the effectiveness of UGS in sustaining biodiversity comparable to natural ecosystems remains debated.

To assess the ecological distinctness of UGS communities from their natural counterparts, we must look beyond measures of local biodiversity. While α-diversity characterizes richness and evenness within a single site, β-diversity quantifies compositional difference between sites, making it particularly valuable for detecting shifts in community assembly along environmental gradients. By partitioning these compositional differences into species turnover (replacement of some species) and nestedness (potential species loss resulting in a subset of a larger community), we can gain deeper insight into the underlying mechanisms of biodiversity change [16]. However, findings are inconsistent across taxa and studies: some show nestedness along urbanization gradients (e.g., carabid beetles, spiders, and ants) [17,18], while others exhibit strong turnover between urban and rural habitats (e.g., true bugs, leafhoppers, and beetles) [19], and patterns can even shift from turnover to nestedness along a steep gradient (e.g., geometrid moths) [20]. This inconsistency suggests that the drivers of community assembly in urban ecosystems may be taxon-, guild-, and context-dependent. Critically, we argue that this inconsistency may reflect a deeper bias: our understanding of urban biodiversity is filtered through an anthropocentric lens. Most existing studies have concentrated on only one trophic level and/or taxa that are directly relevant or appealing to humans (e.g., pollinators, butterflies, and pests) [21,22,23]. In doing so, a human-centered subsample was applied, which does not align with the need to objectively assess biodiversity loss. This leaves the hyperdiverse, abundant, small-sized, taxonomically challenging “neutral” insects [24] or “dark taxa” [25] largely neglected, rendering our assessments of urbanization impacts not just incomplete, but potentially fundamentally biased. Consequently, a critical gap remains in understanding how these overlooked insect communities are reshaped between UGS and adjacent natural habitats.

Diptera (flies and midges) are highly suitable bioindicators of urban ecosystems because: (1) they are among the most species-rich insect orders, exhibiting extraordinary ecological diversity across saprophagous, coprophagous, hematophagous, pollinivorous, phytophagous, predatory, and parasitic lifestyles [26]; (2) while some groups are sensitive to environmental changes, the order also includes numerous widespread synanthropic species [27,28,29]; and (3) they can be effectively sampled using a standardized Malaise trap method: a global study employing this approach revealed that ten of the world’s 20 most species-rich insect families belong to Diptera [30]. Although Malaise traps only subsample the insect communities, when combined with MinION-based DNA barcoding, they provide an efficient, standardized method for less biased, large-scale, specimen-level dipteran diversity assessment [30]. This is essential for evaluating biodiversity through both species’ diversity and their richness, and is also critical for validating identification and confirming unusual findings for a poorly studied fauna.

Numerous studies have highlighted that community stability is maintained by the combined roles of diverse species, from dominant and core species to rare ones [31,32,33]. Consequently, understanding how urbanization filters species to shape these roles, and thus community composition and persistence, is essential for effective conservation. A common approach has been to compare static diversity indices across urban gradients [17,34]. However, such spatial “snapshots” may not capture the temporal duration and richness dynamics that define core and transient species, a dimension critical for understanding true community stability in fluctuating environments. In this study, we move beyond short-term, snapshot assessments by implementing a season-long monitoring program that captures the full phenological cycle of Diptera assemblages. While the present study is based on a single year sampling, the continuous sampling approach provides a comprehensive representation of community dynamics compared to conventional static comparisons.

Here, we address these questions through a spatio-temporal study of Diptera in Haidian District, Beijing. By combining full-season Malaise trap sampling (April to November) with MinION-based DNA barcoding, we test two predictions: (1) urbanization acts as a strong environmental filter such that dipteran community differences between UGS and adjacent shallow mountains will be mainly driven by species replacement (turnover) rather than nested subset formation; and (2) this urban filter operates differentially across trophic guilds. Specifically, we hypothesize that detritivores will primarily exhibit species replacement (turnover) as native taxa are substituted by species adapted to novel anthropogenic resources. In contrast, higher-trophic-level predators and parasitoids will suffer systemic, nested loss due to their inherent sensitivity to habitat simplification and the disruption of complex food webs. We also propose a temporal-abundance framework, which classifies species integrating specimen number and occurrence duration, as a refined lens for diagnosing how communities are architecturally organized.

## 2. Materials and Methods

### 2.1. Study Sites and Sampling Design

**Figure 1:**
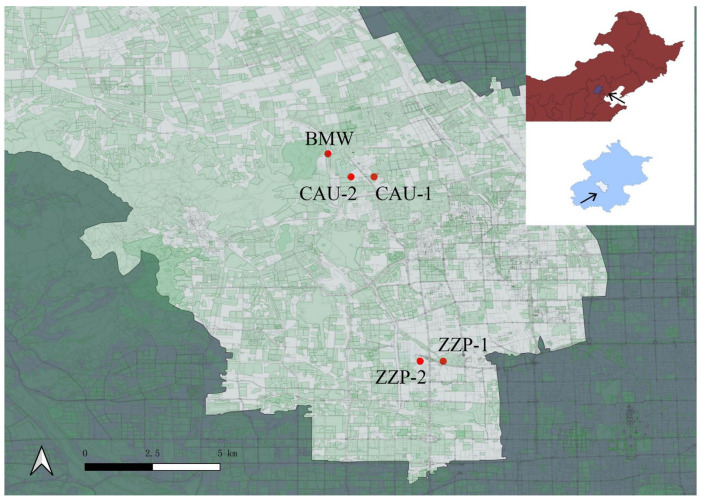
Locations of the five Malaise trap sampling sites in Haidian District, Beijing. The study was conducted across three green space types: two sites (CAU-1 and CAU-2) in residential green space within China Agricultural University; two sites (ZZP-1 and ZZP-2) in Zizhuyuan Park (park green space), and one site (BWM) in Baiwangshan National Forest Park (close-to-natural shallow-mountain green space). Red circles indicate trap locations. Major roads and urban green spaces are shown for spatial reference, while surrounding areas are depicted in gray scale. The inset map situates Haidian District in the northwestern central part of Beijing. The red, blue, and pale green areas and arrows in small maps indicate the location of Beijing in north China and the location of Haidian District in Beijing, respectively; the green areas in the large map indicate urban green space.

This study was conducted in Haidian District, located in the northwestern central Beijing, China. We selected three study sites along a gradient of anthropogenic disturbance: two urban green spaces (UGS), China Agricultural University (CAU, residential green space), and Zizhuyuan Park (ZZP, urban park), and one shallow mountain site, Baiwang Mountain Forest Park (BWM). The maximum distance between any two sites was 12 km, with BWM only 2 km from CAU (Figure 1).

Five Malaise traps (Townes type) were deployed in open areas across these locations. Each trap was equipped with a collection bottle containing approximately 500 mL of absolute ethanol (99.96%). Specific trap placements were as follows: at China Agricultural University: CAU-1 (area with low vegetation richness) and CAU-2 (area with high vegetation richness); at Zizhuyuan Park: ZZP-1 (no pesticide application) and ZZP-2 (area with pesticide application); at Baiwang Mountain Forest Park: BWM (Supplementary Table S1). All fieldwork was conducted with prior permission from the administrations of Baiwang Mountain National Forest Park, Zizhuyuan Park, and China Agricultural University, in compliance with relevant guidelines and regulations.

Sampling was conducted from March to December 2024, with collections made during the first week of each month. Owing to extremely low catch rates (fewer than 10 individuals in total), specimens collected in March and December were excluded from the dataset. Subsequent analysis therefore focused on the eight-month period from April to November. A total of 40 samples were collected. All specimens are deposited in the Entomological Museum of China Agricultural University, Beijing. Dipteran specimens were subsequently sorted for molecular analysis.

### 2.2. Vegetation Survey and Landscape Context

To characterize the habitat surrounding each Malaise trap, a 10 m × 20 m plot was established centered on the trap. Within each plot, all tree individuals with a diameter at breast height (DBH) greater than 3 cm were identified and recorded. Shrubs and herbaceous plants were sampled using a systematic design with five subplots: one at the trap center and one at each of the four plot corners. Shrub subplots were 2 m × 2 m, and herb subplots were 1 m × 1 m. Species, number of individuals, and vegetation density were investigated.

To quantitatively characterize the urbanization gradient, we calculated the proportion of green space within multiple concentric buffers (250 m to 2000 m radius) around each Malaise trap using the Dynamic World V1 (10 m resolution) dataset via Google Earth Engine.

### 2.3. DNA Extraction, Amplification, and Sequencing

All dipteran specimens were processed following the protocols of Srivathsan et al. [35]. This approach was selected for two reasons: (1) the fauna is not well-studied, and DNA barcodes for the majority of the species are not publicly available, making voucher specimens essential for validating identification and confirming unusual findings; and (2) abundance data were required to address our research questions. DNA was extracted using a HotSHOT method, wherein the buffer volume was adapted to the specimen size. Briefly, 10–25 μL of HotSHOT lysis buffer was added to each well of a 96-well plate, incubated at 65 °C for 18 min, followed by heating at 98 °C for 2 min, and subsequently neutralized with an equal volume of neutralization buffer [36]. PCR amplification was performed following the primers and protocols in Zhou et al. [37] with 13 bp tags, and sixteen PCR products per plate were randomly selected and verified on 1% agarose gels.

PCR products (6 μL per well) were pooled and purified using SPRIselect magnetic beads (Beckman Coulter, Brea, CA, USA). DNA concentration was quantified using the ALLSHENG Fluo-200 fluorometer (Allsheng Instruments, Hangzhou, China) with the dsDNA HS assay kit (Invitrogen, Carlsbad, CA, USA). Sequencing libraries were prepared using the SQK-LSK114 ligation sequencing kit (Oxford Nanopore Technologies, Oxford, UK), the NEB#E7546S Ultra II end repair/dA-tailing module (New England Biolabs, Ipswich, MA, USA), and the NEB#E6056S quick ligation module (New England Biolabs, Ipswich, MA, USA). Libraries were sequenced on a MinION Mk1B device (Oxford Nanopore Technologies, Oxford, UK) equipped with R10.4.1 flow cells (Oxford Nanopore Technologies, Oxford, UK) using MinKNOW software v25.03. DNA sequences were demultiplexed and processed using ONTbarcoder v1 [35]. The overall sequencing success rate was 81.75%, with the rate for each sampling site around 80% (Supplementary Table S1).

### 2.4. Molecular Operational Taxonomic Unit (mOTU) Delimitation

Molecular clustering was performed using the software Clustering edlib v1.2.7 [38], which allows sequences of varying lengths to be clustered directly. The “Homologous Unaligned” option was selected with the following parameters: Treat gaps as Missing, Min = 1.0, Max = 5.0. A 3% sequence divergence threshold was applied to delimit molecular operational taxonomic units (mOTUs). Objective mOTU assignments were subsequently obtained using Specimens Sorter (version 0.2) at a 3% threshold, which is widely accepted conservative standard in large-scale Diptera barcoding studies [30,39]. Hereafter, we use the term ‘species’ for simplicity when referring to these mOTUs.

DNA barcodes were then matched against the NCBI NT database using BLAST+ Suite v2.16.0 [40] and against the BOLD public database using the BOLD identification engine [41]. Sequences with ≥95% percentage identity were assigned to the corresponding family. Those with lower homology (<95%) were further examined morphologically by the authors to confirm family-level identification.

For Cecidomyiidae, direct BLAST-based identification was unreliable because the public reference databases (e.g., NCBI) contain many sequences only identified to the family level. Since trophic guilds in this family are conserved at the subfamily level, and multiple guilds are present across the family, we adopted a phylogenetic placement approach to improve taxonomic resolution and infer ecological roles more accurately. We followed the pipeline proposed by Zhou et al. [42] and first compiled all publicly available Cecidomyiidae *COI* barcodes that were identified at least to the subfamily level. After removing duplicates to retain unique haplotypes, we aligned these reference sequences with our own Cecidomyiidae sequences. A maximum likelihood tree was then constructed using IQ-TREE v.2.4.0 [43] under the GTR model [44]. Subfamily- to genus-level identifications were assigned based on well-supported monophyletic clustering of our sequences with named reference clades.

### 2.5. Partition of the Species Assemblage into Trophic and Abundance-Persistence Groups

Diptera specimens were classified into three primary trophic guilds: detritivores, herbivores, and predators/parasitoids (including hematophagous) following Marshall et al. [26]. Detritivores encompass saprophagous, coprophagous, and mycetophagous species that function as decomposers in natural ecosystems. Herbivores include all species that feed on primary producers, such as living plant tissues. The predators/parasitoids guild comprises species that obtain nutrition from other animals, either through predation, parasitism, or hematophagy.

Furthermore, the entire species assemblage was partitioned into four distinct ecological categories based on their temporal persistence (duration) and total relative abundance. We set the critical relative abundance threshold at 1.0% to distinguish dominant from subordinate taxa, and a temporal threshold of four months of presence. Based on these dual thresholds, species were defined as follows: Core species: Species with a relative abundance of ≥1.0% and a temporal duration of ≥4 months. Seasonal species: Species with a relative abundance of ≥1.0% but a temporal duration of <4 months. Background species: Species with a relative abundance of <1.0% and a temporal duration of ≥4 months. Rare species: Species with a relative abundance of <1.0% and a temporal duration of <4 months.

### 2.6. Diversity Analyses

Prior to analysis, all data were organized in Microsoft Excel (version 2403). All subsequent analyses were conducted in R v4.3.3 [45]. Sample completeness curves were generated using the iNEXT package v3.0.1 [46] through interpolation and extrapolation to assess sampling coverage.

Beta diversity for species and functional groups was conducted based on the Baselga-Sørensen framework [16] to partition total dissimilarity into turnover and nestedness components. To quantify community differentiation, we decomposed total Sorensen dissimilarity (β_sor_) into two additive components: turnover (β_sim_) and nestedness-resultant dissimilarity (β_nes_), following the framework described by Baselga [47]. All computations were performed using the adespatial package v0.3.28 [48]. Significant differences between groups were determined by Kruskal–Wallis non-parametric tests, with results visualized using beta diversity decomposition plots.

To explore temporal and spatial patterns in species composition, Bray–Curtis dissimilarity matrices were constructed from species abundance data. Non-metric multidimensional scaling (NMDS) was applied to ordinate the Diptera communities, with stress values used to assess model fit. Permutational multivariate analysis of variance (PERMANOVA; function adonis2) was employed to test for significant compositional differences among predefined groups. To test the PERMANOVA assumption of homogeneity of multivariate dispersions, we used the betadisper function in the vegan package v2.6.10 [49].

To determine whether the observed compositional variations were driven by dispersal limitation or environmental filtering, the distance–decay relationship of community similarity was evaluated using Mantel tests. First, a geographic distance matrix was constructed using the pairwise spatial distances among the five sampling sites. Community dissimilarity was quantified using overall β-diversity, which was subsequently partitioned into two distinct components: species turnover (spatial replacement) and nestedness (species loss or gain). Mantel tests were then performed to assess the statistical correlation between the geographic distance matrix and each of the three dissimilarity matrices (total β-diversity, turnover, and nestedness). The significance of the Mantel statistic (r) was tested using 999 permutations.

To evaluate the influence of vegetation on Diptera community structure, we performed correlation analyses between vegetation metrics and insect diversity across the five sampling sites. To account for differences in sampling area and vertical stratification, vegetation metrics were calculated separately for tree, shrub, and herb layers: (1) Layer-specific Plant Richness, defined as the number of vascular plant species identified within the respective sampling layer at each site; (2) Layer-specific Plant Density, calculated as the number of plant individuals per unit area (individuals m^−2^) for each layer, which standardizes for differing plot sizes (trees: 200 m^2^; shrubs: 4 m^2^; herbs: 1 m^2^). For the Diptera communities, we analyzed the species richness of the total assemblage as well as the richness of three distinct functional guilds (herbivores, detritivores, and predators/parasitoids). Given the small sample size (N = 5), we used Pearson correlation coefficients (R) to quantify the strength and direction of these relationships. Simple linear regression models were further employed to visualize trends and to calculate the coefficient of determination (R^2^).

## 3. Results

### 3.1. Urbanization Gradient Validation

To validate the assumed disturbance gradient, we quantified green space coverage around each sampling site. Based on linear regression between landscape metrics and species richness, the 500 m scale was identified as the most representative landscape context (R^2^ = 0.695). Green space coverage decreased from 69.7% at BWM to an average of 3.8% at ZZP and 2.3% at CAU, confirming a stark environmental gradient (Supplementary Table S2).

### 3.2. Species Composition

A total of 6762 Diptera specimens were collected from 45 Malaise trap samples across five sites from April to November 2024. Successful sequencing was achieved for 5528 specimens, representing 39 families and 686 putative species (Supplementary Table S3, Supplementary Figure S1). Sample completeness curves indicated coverage exceeded 75% at all five sites and approached an asymptote (Supplementary Figure S2). Successfully sequenced specimens were used for the following analyses.

Species richness and abundance at the five sampling sites, ranked from highest to lowest richness, were: BWM (Baiwang Mountain Forest Park; 1571 specimens, 333 species), ZZP-1 (Zizhuyuan Park, no pesticide application; 1344 specimens, 239 species), CAU-2 (China Agricultural University, area with high vegetation richness; 1302 specimens, 223 species), ZZP-2 (Zizhuyuan Park, area with pesticide application; 810 specimens, 173 species), and CAU-1 (China Agricultural University, area with low vegetation richness; 501 specimens, 109 species) (Supplementary Figure S3).

### 3.3. β-Diversity and Community Similarity

Given that a substantial portion of species were represented by few individuals, and such rare species can disproportionately influence ordination and diversity metrics [50,51], we performed two parallel analyses: one with and one without rare species (species with <8 individuals) to assess the robustness of observed patterns.

**Figure 2:**
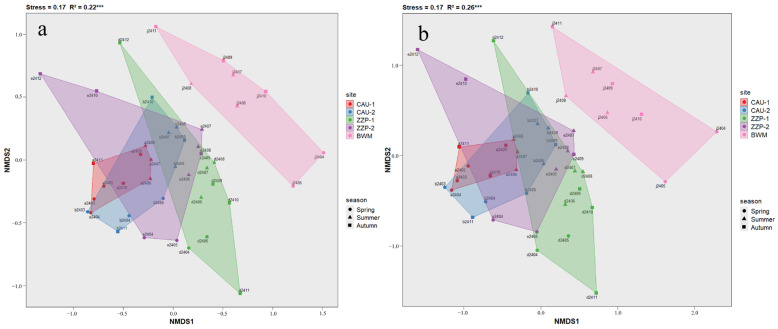
Non-metric multidimensional scaling (NMDS) of Diptera community composition across five sites. Ordination is based on a Bray–Curtis dissimilarity matrix of species abundances. (**a**) Analysis of the full community. (**b**) Analysis after removal of rare species (those represented by fewer than 8 individuals, comprising 25% of all species). Each point represents a Malaise trap sample, colored by site (CAU-1, CAU-2, ZZP-1, ZZP-2, BWM). Polygons are used to group samples by site. Stress values are provided for each ordination. Use PERMANOVA (function adonis2) for significance test. ***: *p* ≤ 0.001.

Non-metric multidimensional scaling (NMDS) of the full community revealed a clear separation between sites (stress = 0.17; Figure 2). Samples from the shallow mountain site (BWM) formed a distinct cluster, separate from the overlapping cluster containing all four urban sites (CAU-1, CAU-2, ZZP-1, ZZP-2) (Figure 2a). Permutational multivariate analysis of variance (PERMANOVA) confirmed that site identity explained a significant portion of compositional variation (R^2^ = 0.22, *p* < 0.001). The assumption of homogeneity of multivariate dispersions was validated using PERMDISP (F = 2.22, *p* = 0.079 > 0.05), indicating that the observed community differentiation reflects a true shift in species composition rather than an artifact of unequal within-group variance. This spatial pattern remained virtually unchanged after removing the rare species tail (Figure 2b).

**Figure 3:**
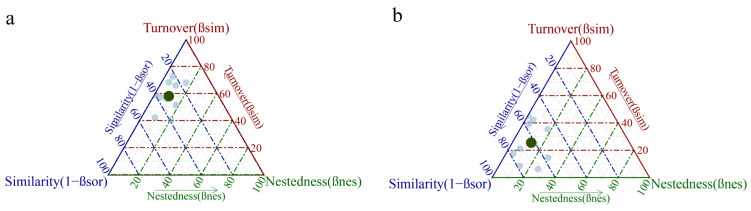
Partitioning of β-diversity (Baselga–Sørensen framework) among the five sampling sites. (**a**) Total dissimilarity (βsør) partitioned into species turnover (β_sim_) and nestedness (β_nes_) components for the full species community. (**b**) Partitioning performed after the exclusion of the rare species tail (species with <8 individuals, constituting 25% of all species). Light-colored dots indicate site pairs; dark-colored dots represent mean values.

Beta diversity partitioning (Baselga-Sørensen framework) for the full community revealed substantial compositional heterogeneity across the study area (βsør: 0.51–0.84). Dissimilarity was most pronounced between the shallow mountain site and the urban sites (βsør > 0.73), while urban site pairs showed lower dissimilarity (βsør = 0.51–0.70). Among all comparisons, the turnover component (β_sim_) was the dominant driver of beta diversity, exceeding nestedness (β_nes_) by a factor of 1.97 to 41.08 (Supplementary Table S4, Figure 3a). Excluding the rare species tail, comprising 25% of the putative species, resulted in a substantial reduction in total beta diversity (βsør decreased by 36–63%) and its turnover component (β_sim_ decreased by 35–84%). However, species turnover remained the dominant process structuring beta diversity. This pattern was most pronounced in comparisons involving the shallow mountain site (BWM). Notably, while the β_sim_/β_nes_ ratio decreased in all urban-urban comparisons after excluding rare species, it substantially increased for the BWM_CAU-2 and BWM_ZZP-2 pairs. Consequently, the dominance of turnover over nestedness became even more extreme in these specific mountain-urban comparisons, with β_sim_/β_nes_ ratios reaching 2.01 to 141.38 (Supplementary Table S4, Figure 3b). Nestedness became the dominant component for half of the urban site pairs when the rare species tail was excluded (Supplementary Table S4).

### 3.4. Distance and Community Similarity

The Mantel test showed no significant correlation between geographic distance and community dissimilarity (βsør: Mantel r = 0.159, *p* = 0.283), indicating that a distance–decay relationship was not detectable within the sampled spatial range (~2–12 km) (Supplementary Figure S4).

### 3.5. Trophic Guild Composition and Beta Diversity Patterns

**Figure 4:**
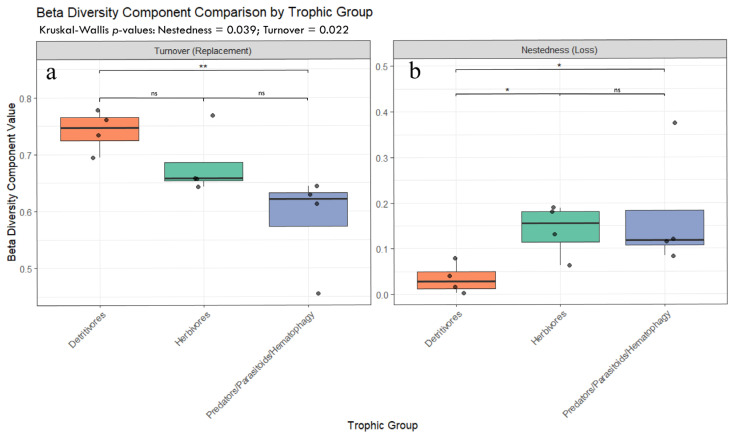
Partitioning of β-diversity among trophic guilds between the shallow mountain (BWM) and urban sites. (**a**) Turnover component (β_sim_) and (**b**) nestedness component (βnes) for each guild. Values represent pairwise comparisons between BWM and each of the four urban sites. Differences among guilds were tested with the Kruskal–Wallis test; significant pairwise differences (Dunn’s post hoc test with Bonferroni correction, ns: *p* > 0.05; *: 0.01 < *p* ≤ 0.05; **: 0.001 < *p* ≤ 0.01.) are indicated by asterisks. Boxplots show the median, interquartile range, and 5–95 percentiles.

Trophic guild classification results are shown in Supplementary Table S5. Beta diversity partitioning across trophic guilds between the shallow mountain site and urban sites revealed that species turnover (β_sim_) was the dominant component for all guilds, with median values significantly higher than nestedness (β_nes_) (Figure 4; Supplementary Table S6). However, the magnitude of these components differed significantly among guilds (Kruskal–Wallis tests, *p* < 0.05 for both β_sim_ and β_nes_). Post hoc tests showed that detritivores exhibited significantly higher turnover (β_sim_) than predators/parasitoids (Figure 4a). Consequently, detritivores had a significantly lower nestedness (β_nes_) than both herbivores and predators/parasitoids, which had similar nestedness values (Figure 4b).

### 3.6. Exploratory Analysis of Vegetation Drivers

Vegetation density (a composite measure of cover and abundance) varied across sites, with the following descending order: BWM > ZZP-1 > CAU-2 > CAU-1 > ZZP-2 (see Supplementary Table S7 for full details). Vegetation structure exhibited distinct vertical stratification across the sampling sites, with standardized densities and species richness varying significantly (Table S2). We performed exploratory correlation analyses to identify potential associations between vegetation and Diptera functional guilds (Supplementary Figure S6).

Our results indicated positive trends between vegetation complexity and certain guilds. Specifically, herb layer density showed a strong positive association with the species richness of herbivorous Diptera (R^2^ = 0.91, *p* = 0.012; Supplementary Figure S7c). Herb layer density also showed positive but non-significant trends with the richness of detritivores and predators/parasitoids (Supplementary Figure S7a,b).

Tree species richness was significantly and positively correlated with the richness of predators and parasitoids (R^2^ = 0.79, *p* = 0.043; Supplementary Figure S7d). However, tree richness did not show significant correlations with the richness of herbivores or detritivores (Supplementary Figure S7e,f). Herb layer density and tree richness did not show clear associations with detritivores, and shrub layer metrics generally exhibited weaker, non-significant relationships with all functional guilds.

However, these statistical values should be interpreted as indicative trends rather than conclusive evidence due to the potential for overfitting in a small dataset (Supplementary Figure S6).

### 3.7. Species Composition Patterns Based on Abundance and Temporal Persistence

**Figure 5:**
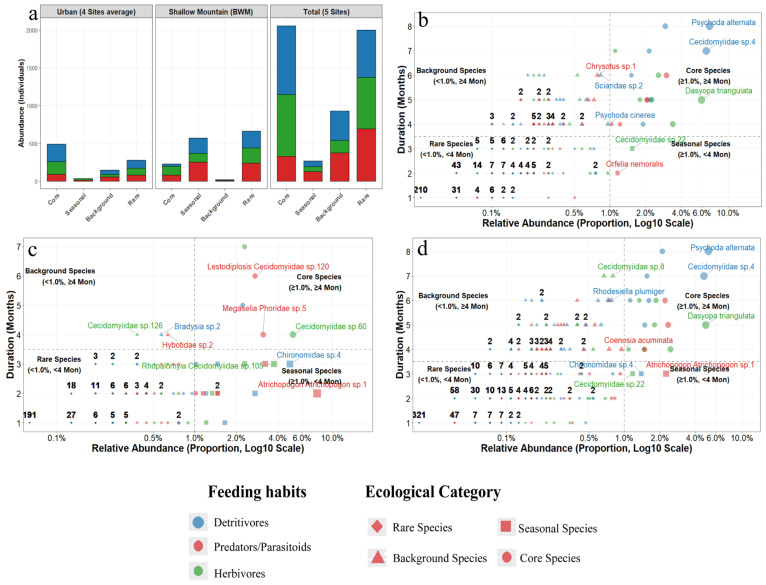
Spatiotemporal dynamics and functional structure of Diptera communities across an urban-natural gradient. (**a**) Standardized absolute abundance of individuals within each ecological quadrant across the urban sites, the shallow mountain site (BWM), and the total regional pool. To eliminate the bias of sampling effort, the abundance for the urban group is averaged across its four constituent sites. (**b**–**d**) Ecological classification of Diptera species based on total relative abundance and temporal persistence for (**b**) the four urban sites combined (CAU-1, CAU-2, ZZP-1, ZZP-2), (**c**) the shallow mountain site (BWM), and (**d**) the entire regional assemblage (all five sites combined). Species are categorized based on a relative abundance threshold of 1.0% and a temporal duration threshold of 4 months: Core (abundance ≥ 1.0%, duration ≥ 4 months), Seasonal (abundance ≥ 1.0%, duration < 4 months), Background (abundance < 1.0%, duration ≥ 4 months), and Rare (abundance < 1.0%, duration < 4 months). Points and bars are colored by trophic guild: blue = detritivores, green = herbivores, red = predators/parasitoids/hematophagous. Dashed lines indicate classification thresholds. The five most abundant species in each category are labeled. Numeric labels on points indicate the number of overlapping species sharing identical coordinates.

Standardized absolute abundance revealed stark contrasts in community size and architecture between the shallow mountain site (BWM) and urban sites (CAU, ZZP) (Figure 5a). The community at BWM was supported by a massive reservoir of Rare and Seasonal Species (Figure 5a,b). Despite having the highest overall species richness and absolute abundance, it harbored only five Core Species, none exceeding 100 individuals.

In contrast, urban communities exhibited a drastically reduced overall abundance and were disproportionately structured around a highly limited set of Core Species (Figure 5a,c). Although few in number, these Core Species (predominantly detritivores) monopolized the total community abundance in urban habitats (Figure 5c; Supplementary Figure S5). Urban areas also harbored more background species (low abundance, high persistence) than BWM, whereas Seasonal Species were significantly fewer (Figure 5a,c).

This divergence in community structure was mirrored by trophic composition (Supplementary Table S5). All four urban sites exhibited a higher proportion of detritivores (range: 34.97–64.07%; Supplementary Figure S5) compared to BWM (31.83%; Figure 5b). Conversely, the proportion of predators/parasitoids was notably higher at BWM (37.94%) than at any urban site (range: 17.45–30.65%; Supplementary Figure S5). Herbivores proportion showed no consistent difference between BWM and urban sites, with the highest value observed at CAU-2 and the lowest at CAU-1.

## 4. Discussion

### 4.1. Composition of the Urban Diptera Fauna

Our study reveals that the Diptera family-level composition in the shallow mountain site (BWM) largely aligns with global patterns observed in protected areas [30], while the urban sites deviate (Supplementary Figure S1). This discrepancy suggests an urban environmental filter that differentially selects for certain families (e.g., increased prominence of Agromyzidae, Drosophilidae, Mycetophilidae, and Calliphoridae) while reducing the representation of others (e.g., Dolichopodidae, Psychodidae, and Sphaeroceridae). Notably, even within families generally filtered against in cities, highly synanthropic species (e.g., *Psychoda alternata* and *Clogmia albipunctata*; *Psychodidae*) can thrive, highlighting species-level adaptation.

### 4.2. Urbanization Drives Species Replacement, Not Subset Formation, Between Urban Habitat and Adjacent Shallow Mountains

Our β-diversity analysis reveals a striking pattern: cities are not passive recipients of biodiversity from nearby natural areas, but powerful ecological filters that actively reshape communities. Rather than acting as permeable sinks that receive subsets of the surrounding fauna, the urban environment drives extensive species replacement (Figure 2 and Figure 3, Supplementary Table S3). Across all mountain-urban comparisons, the turnover component (β_sim_) overwhelmingly dominated β-diversity, and this signal became even stronger once rare species were excluded (Figure 2 and Figure 3, Supplementary Table S3). This demonstrates that urban and shallow mountain dipteran assemblages are built from fundamentally different sets of common species.

The prevalence of species replacement over nested loss indicates that urbanization does not simply erode diversity, but actively reassembles it. Cities host communities that are compositionally distinct from those of adjacent natural habitats, even at short spatial distances. This pattern aligns with findings from other studies, where urban habitats consistently favor turnover rather than the hierarchical filtering of regional species pools [19,52,53,54].

The urban filter appears to operate in stages. Initial habitat conversion eliminates strict forest specialists, sharply narrowing the pool of potential colonists. The urban environment then imposes a second, more selective filter, favoring disturbance-tolerant, often synanthropic, even some exotic species. These taxa frequently reach high abundances and may gain competitive advantages and may further exclude any remaining generalists derived from the natural habitats [55,56,57]. The result is not a depauperate shadow of the surrounding fauna, but a novel and distinctly urban assemblage.

A critical consideration is the role of geographical distance and connectivity. While some studies emphasize their importance for diversity within urban matrices [58,59], our findings suggest its influence here is limited. Despite the close proximity (~2 km) between the shallow mountain and the nearest urban site, we observed extreme turnover, whereas greater distances between urban sites themselves yielded increased nestedness. This stark contrast, together with the weak distance–decay relationships detected by Mantel tests, highlights that the dominant force structuring these communities is not isolation-by-distance, but the qualitative environmental filter imposed by the urban matrix. However, it is important to note that the lack of a significant distance–decay relationship might also be a function of the relatively small spatial scale of our study. At such scales, environmental filtering appears to be the dominant driver of community structure, overriding the effects of isolation-by-distance. This is consistent with previous studies suggesting that environmental gradient exerts much stronger selection pressures on insect communities than local spatial positioning.

The strength of this filter is further evidenced by the biotic homogenization observed within the urban matrix itself. The increased relative contribution of nestedness (β_nes_) among urban sites after rare species removal (Figure 3b) suggests that internal differences are primarily due to species loss from a shared urban-adapted pool, rather than further compositional turnover. Therefore, our results underscore a dual conservation significance. First, they highlight the exceptional value of adjacent natural habitats like shallow mountains, which maintain a unique and diverse species pool fundamentally distinct from that of the surrounding urban matrix. Second, they reveal the intense filtering strength of the current urban environment, which creates a strong compositional disjunction, even over short spatial scales.

### 4.3. Urbanization Imposes a Modular Filter on Trophic Guilds

Our trophic-level analysis reveals that the urban filter does not act as a monolithic or uniform pressure across the entire Diptera community. Instead, it operates as a “modular filter”—a system of partitioned filtering mechanisms where distinct functional guilds (or “modules”) follow independent assembly rules and respond to divergent environmental drivers. This modularity results in the asymmetric restructuring of community components, with direct consequences for ecosystem function stability (Figure 4).

The first module consists of detritivores (saprophagous or mycophagous), which exhibited the highest species turnover (β_sim_) and the lowest nestedness (β_nes_) between urban and shallow mountain sites (Figure 4). This pattern of strong species replacement points to a fundamental shift in resource base. In natural habitats, heterogeneous substrates such as forest leaf litter and diverse fungal resources [60,61]. Urban environments, by contrast, provide predictable, anthropogenic subsidies, organic waste, compost, drain films, and mulch [62,63,64]. The decomposition function in cities is thus maintained not by a subset of the natural decomposer pool, but by a reconstituted, urban-adapted assemblage [65]. These continuous resources also buffer against seasonal fluctuations, supporting less temporally constrained insect assemblages [66,67]. This explains why detritivores constitute the majority of core species in our urban temporal-abundance framework (Figure 5b and Figure S4). Supporting this resource-based interpretation, our exploratory analysis suggested that detritivore diversity was largely decoupled from metrics of living plant vegetation (Figure S7b,e). While based on a limited number of sampling sites (N = 5), this lack of association implies that urban decomposer communities are structured primarily by anthropogenic subsidies rather than by standing green biomass.

In contrast, the predators/parasitoids module exhibited significantly higher nestedness and the lowest turnover (Figure 4), indicating a greater degree of species loss without equivalent urban-adapted replacement. This aligns with the trophic rank hypothesis, which posits that higher trophic levels are more sensitive to disturbance due to their dependence on prey diversity and habitat complexity [68,69,70]. The loss of prey diversity in simplified urban habitats, coupled with reduced structural complexity, likely results in a cascading loss of natural enemies that cannot be compensated by colonization from the surrounding landscape. This sensitivity was further indicated by a positive exploratory trend between predator/parasitoid richness and tree species richness (R^2^ = 0.79, Figure S7d). Although the small sample size (N = 5) requires cautious interpretation, this indicative relationship suggests that for higher trophic levels, the structural and taxonomic complexity of the canopy may provide critical niche diversity and alternative resources.

The herbivores module presented an intermediate pattern. While acknowledging the limitations of our small dataset, our exploratory results showed that herbivore diversity followed a strong positive trend with herb layer density (R^2^ = 0.91, Figure S7c) rather than plant species richness. This aligns with a “More Individuals” mechanism [15,71], where resource availability drives species richness. This strong biomass-dependent relationship aligns with the high species turnover (β_sim_) observed for this guild, indicating that urban herbivore assemblages are shaped primarily by the availability and type of understory vegetation rather than by nested loss from a regional species pool. These results should be interpreted as hypothesis-generating rather than as formal inferential tests.

These guild-specific assembly mechanisms—operating as independent ecological modules—call for multi-targeted conservation strategies. Supporting herbivore diversity requires promoting dense understory vegetation. Mitigating predator loss depends on maintaining diverse tree canopies. And fostering diverse decomposer assemblages requires ensuring the provision and retention of heterogeneous organic matter such as leaf litter and woody debris. But diagnosing which guild is most impacted in a given urban setting, and whether conservation interventions are working, requires a standardized framework for assessing community health. Such a framework must capture not only which species are present, but how communities are structured along axes of abundance and temporal persistence. This is where our temporal-abundance approach provides a critical tool.

Our landscape analysis provides quantitative support for the urbanization gradient. The high explanatory power of green cover at the 500 m scale (R^2^ = 0.695, *p* = 0.079) suggests that the reduction in habitat area acts as a deterministic environmental filter rather than a purely stochastic process. Although the *p*-value is marginally above 0.05 due to the limited number of sampling sites, the steep decline in richness alongside the reduction in green cover (from ~70% to <5%) strongly indicates that urbanization intensity is a primary driver of community simplification. Furthermore, the variation in richness observed among sites with similarly low landscape green cover (e.g., CAU-1 vs. CAU-2) suggests that once regional urbanization reaches a certain threshold, local habitat quality and vegetation structure may become crucial secondary filters.

Admittedly, our study design reflects a contrast between one large natural fragment (BWM) and four relatively small urban green space patches. While our GEE-based metrics verify this as a robust urbanization gradient, we acknowledge that the effects of habitat area (island biogeography) and urbanization intensity (environmental filtering) are often intertwined. The current sampling layout primarily captures the extreme ends of this spectrum. Future studies incorporating a larger number of intermediate-sized patches and more replicates across the urban matrix are needed to fully decouple the relative contributions of patch size versus matrix quality on Diptera community assembly.

### 4.4. Toward a Diagnostic Framework: Abundance and Temporal Persistence as Axes of Community Architecture

Our temporal-abundance framework, which classifies species by both specimen number and occurrence duration (Figure 5), provides a refined lens for diagnosing how communities are architecturally organized, which is essential for detecting guild-specific degradation.

Applying this framework reveals that the urban and shallow mountain communities are built on fundamentally different architectures. The shallow mountain community structure is characterized by a long “tail” of Rare Species and the highest number of Seasonal Species, with Core Species being minimal, but represents all three trophic guilds. In stark contrast, the urban community structure is reorganized around a limited set of Core Species that achieve high abundance and long persistence and are dominated by detritivores.

The diagnostic power of our temporal-abundance framework, however, depends on establishing a baseline. Despite the insights provided, it is important to note that our study is based on a single year of data. Long-term monitoring is crucial for accurately defining core species assemblages and understanding inter-annual variability in community structure [72,73,74]. Moreover, in the present study we used provisional cutoffs for abundance (1.0%) and duration (four months) based on the structure of our data. These should not be considered recommended universal cutoff values. We encourage future studies to explore threshold sensitivity for different taxonomic groups and environmental contexts.

The framework itself establishes the essential methodology for such future monitoring. By tracking shifts in core, seasonal, and rare species over time, comparing a local reference with its closely located urban counterpart, this approach will be pivotal for establishing ecological baselines and diagnosing which guild is most impacted in a given urban setting. Ultimately, it offers a powerful tool for assessing ecological integrity and guiding urban planning and restoration strategies for biodiversity conservation.

A caveat of our study is the reliance on a single-year dataset, which precludes the assessment of interannual variability. Year-to-year fluctuations in climate can significantly impact insect richness and abundance. However, the filtering pressure exerted by the physical urban matrix—such as impervious surfaces and habitat fragmentation—remains relatively stable over time. Thus, while absolute diversity indices may vary annually, the direction and mechanism of the “modular filter” across trophic guilds are likely consistent. Future long-term monitoring is essential to decouple the interactions between climate variability and urbanization intensity and to verify the interannual stability of the assembly rules identified here.

## 5. Conclusions

Our study confirms both predictions about how urbanization reshapes insect communities. First, we demonstrate that cities are not passive sinks receiving subsets of nearby natural fauna, but powerful environmental filters that drive species replacement (turnover) rather than nested species loss, despite close geographic proximity. Therefore, while urban green spaces are valuable, they cannot substitute for nearby natural habitats, which maintain fundamentally distinct and architecturally complex assemblages. Second, we show that the urban filter operates differentially across trophic guilds: detritivore assemblages are reconstituted around anthropogenic resources, exhibiting high turnover and no correlation with vegetation; predator assemblages suffer nested species loss linked to reduced tree canopy diversity; and herbivores occupy an intermediate position, with diversity tightly coupled to understory density. This highlights the need for guild-specific conservation strategies and establishes a diagnostic framework for assessing community composition. Extending beyond these predictions, our temporal-abundance framework, which classifies species by both specimen number and occurrence duration, provides a refined lens for diagnosing how communities are architecturally organized, an approach essential for detecting guild-specific degradation. With long-term monitoring to establish robust baselines, this framework offers a pathway for evaluating ecological integrity and guiding biodiversity-sensitive urban planning.

## Data Availability

The original contributions presented in this study are included in the Supplementary Materials. Further inquiries can be directed to the corresponding authors.

## Supplementary Materials

The following supporting information can be downloaded at: https://www.mdpi.com/article/10.3390/biology15110865/s1. Figure S1. Species and specimen composition of Diptera families across five sampling sites. Families are sorted in descending order of total species richness. Bars represent the number of species (richness, left *y*-axis), and points connected by a line represent the number of specimens (abundance, right *y*-axis) for each family. Sites are differentiated by color, as shown in the legend. Figure S2. Sample completeness curves for Diptera communities across five sampling sites. Curves show estimated sample coverage (proportion of the community represented) as a function of sampling effort (number of individuals sequenced). All curves approach an asymptote above 75% coverage, indicating that sampling effort was sufficient to adequately characterize the local species composition at each site. Figure S3. Relative contribution of Diptera families to total community diversity at each sampling site. (a) Relative species richness (proportion of total species per site). (b) Relative abundance (proportion of total specimens per site). Families are represented by colors as shown in the legend. Bars are grouped by site (CAU-1, CAU-2, ZZP-1, ZZP-2, BWM). Figure S4. Correlation between geographic distance and community dissimilarity (Sørensen index). The relationship is partitioned into (a) total β-diversity, (b) species turnover, and (c) nestedness-resultant components. Mantel r and P-values are indicated within each panel. Figure S5. Ecological classification of Diptera species for each urban site. (a) CAU-1, (b) CAU-2, (c) ZZP-1, (d) ZZP-2. Species are categorized based on a relative abundance threshold of 1.0% and a temporal duration threshold of 4 months: Core (abundance ≥ 1.0%, duration ≥ 4 months), Seasonal (abundance ≥ 1.0%, duration < 4 months), Background (abundance < 1.0%, duration ≥ 4 months), and Rare (abundance < 1.0%, duration < 4 months). Points and bars are colored by trophic guild: blue = detritivores, green = herbivores, red = predators/parasitoids/hematophagous. Dashed lines indicate classification thresholds. The five most abundant species in each category are labeled. Numeric labels on points indicate the number of overlapping species sharing identical coordinates. Figure S6. Correlation heatmap of vegetation structure and Diptera diversity. The heatmap displays the Pearson correlation strength (R) between stratified vegetation parameters (Tree, Shrub, and Herb layers) and major Diptera functional guilds. Red indicates positive correlation, while blue indicates negative correlation. Numbers within cells represent the correlation coefficients. Figure S7. Impact of herb layer density and tree richness on Diptera functional guilds. Scatter plots show the relationships between key vegetation drivers and insect richness across five sampling sites. (a–c) The influence of herb layer density on (a) predators/parasitoids, (b) detritivores, and (c) herbivores. (d–f) The influence of tree layer richness on (d) predators/parasitoids, (e) detritivores, and (f) herbivores. Each plot includes a linear regression line (solid) with a 95% confidence interval (shaded). Individual sampling sites are color-coded. Table S1. Information of Malaise Trap Sampling Sites and sequencing success rate. Table S2. Species richness of Diptera trophic guilds across sampling sites. Table S3. Raw specimen data. Table S4. Partitioning of the pairwise Baselga-Sørensen index (βsør) into species replacement (βsim) and nestedness (βnes) components. Table S5. Species and trophic guild classification. Table S6. Species richness and individual abundance of Diptera trophic guilds across sampling sites. Table S7. Vegetation survey information for Malaise trap sites.
