# Supplementary material for: Urban Filter vs. Natural Refuge: Divergent Diptera Community Assembly Mechanisms—Evidence from Beijing, China
Source: Biology (Basel). 2026 May 30;15(11):865. doi: 10.3390/biology15110865 (PMC13255993; doi:10.3390/biology15110865)
Supplement: Supplementary file 1 [file biology-15-00865-s001.zip › Beijing_urban_fly_diversity_Supplementary_Figures-submit.pdf]

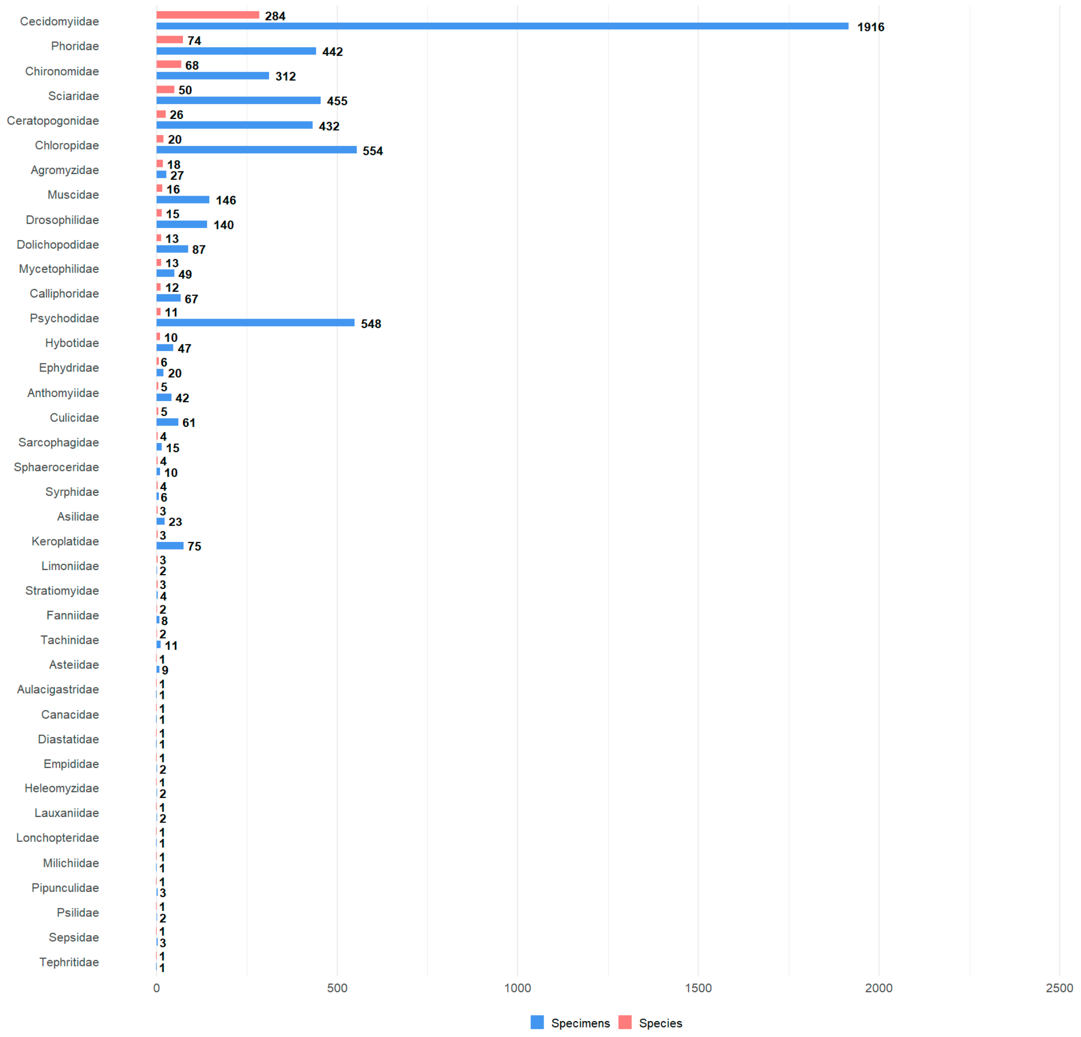

Figure S1. Species and specimen composition of Diptera families across five sampling sites. Families are sorted in descending order of total species richness. Bars represent the number of species (richness, left y-axis), and points connected by a line represent the number of specimens (abundance, right y-axis) for each family. Sites are differentiated by color, as shown in the legend.

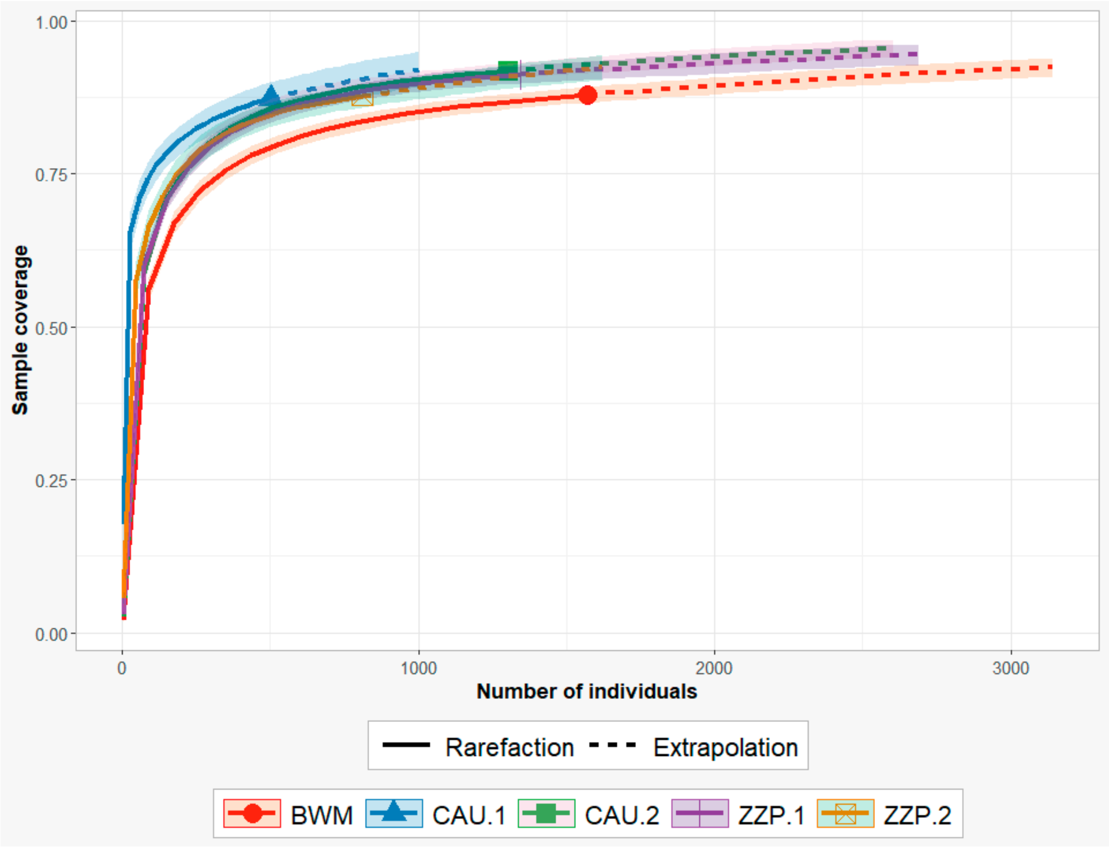

Figure S2. Sample completeness curves for Diptera communities across five sampling sites. Curves show estimated sample coverage (proportion of the community represented) as a function of sampling effort (number of individuals sequenced). All curves approach an asymptote above 75% coverage, indicating that sampling effort was sufficient to adequately characterize the local species composition at each site.

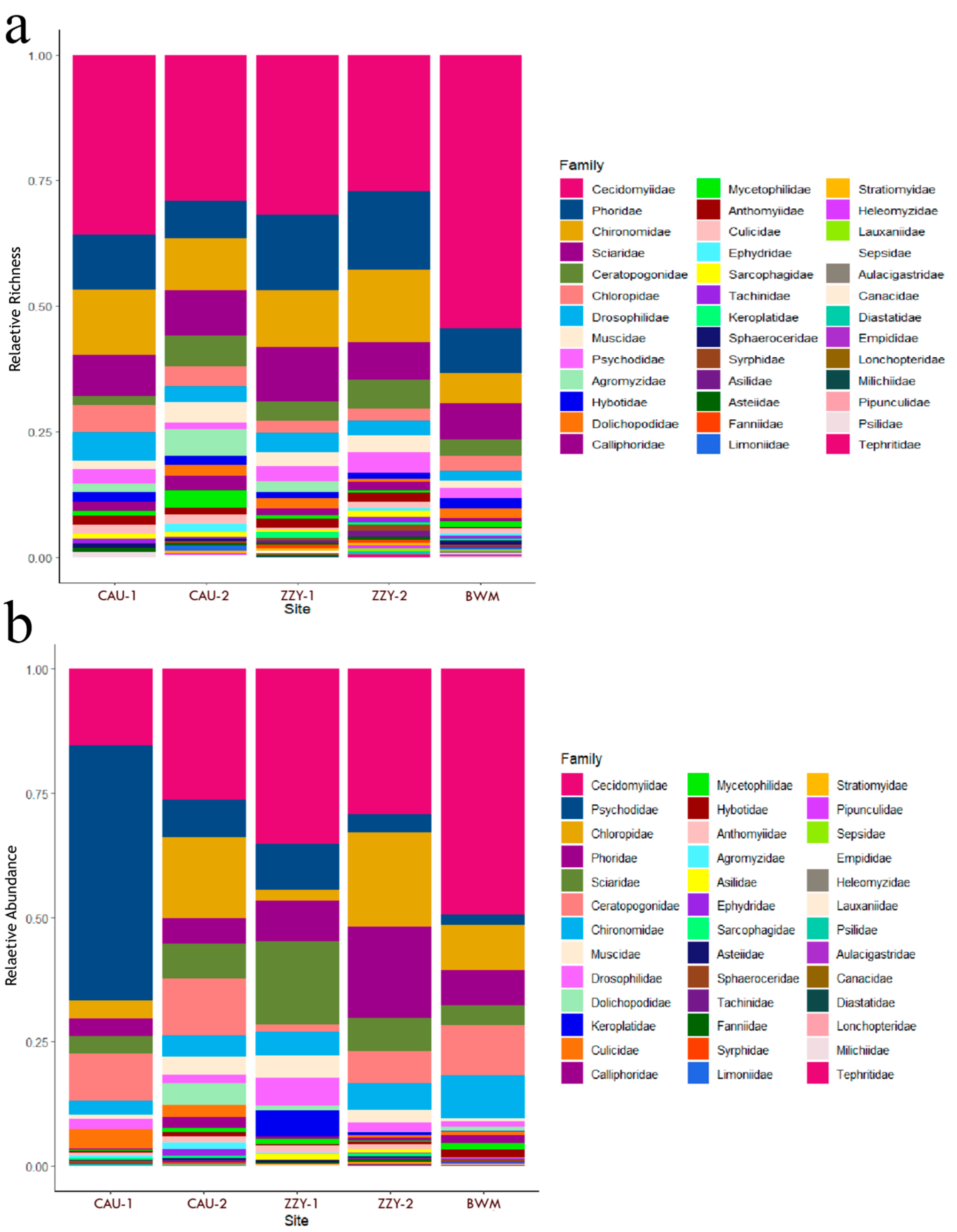

Figure S3. Relative contribution of Diptera families to total community diversity at each sampling site. (a) Relative species richness (proportion of total species per site). (b) Relative abundance (proportion of total specimens per site). Families are represented by colors as shown in the legend. Bars are grouped by site (CAU-1, CAU-2, ZZP-1, ZZP-2, BWM).

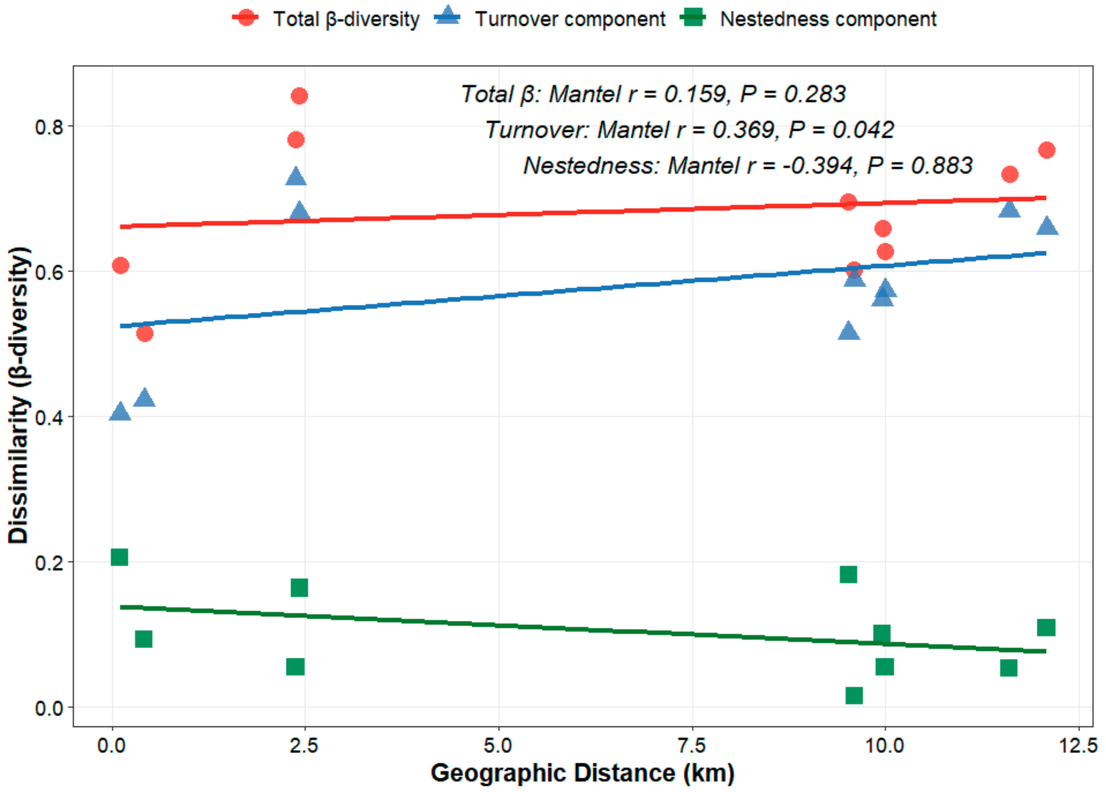

Figure S4. Correlation between geographic distance and community dissimilarity (Sørensen index). The relationship is partitioned into (a) total β-diversity, (b) species turnover, and (c) nestedness-resultant components. Mantel r and P-values are indicated within each panel.

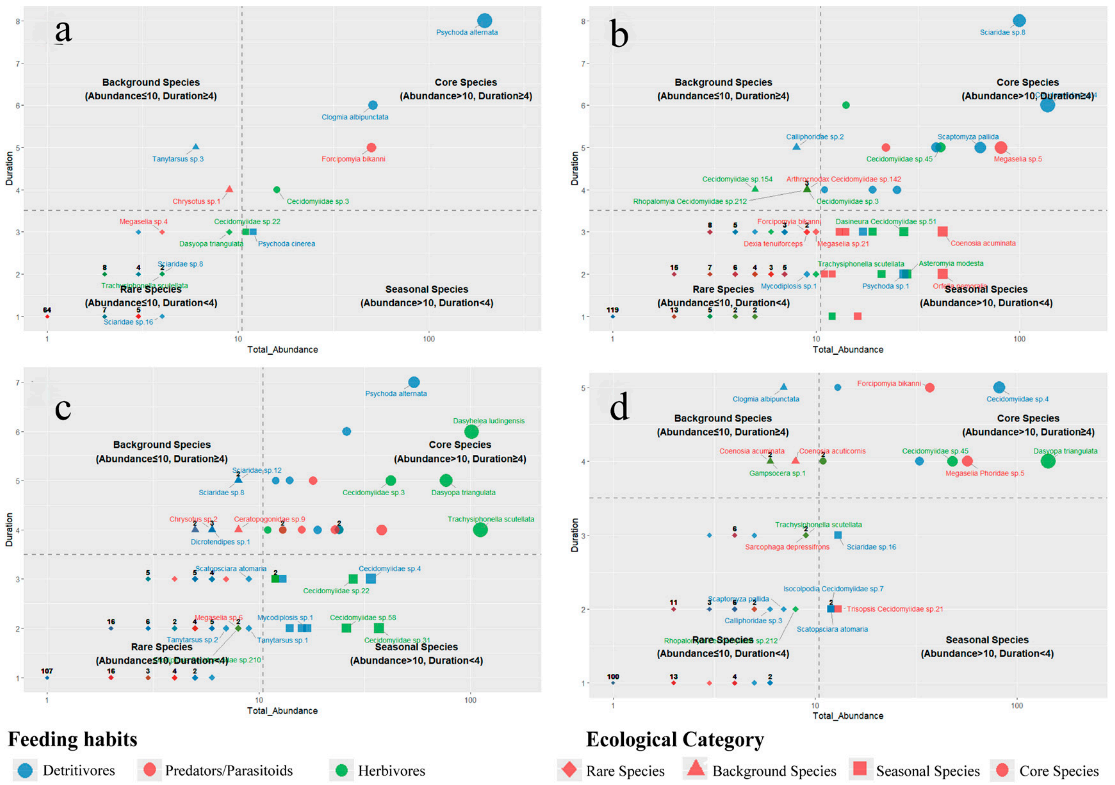

Figure S5. Ecological classification of Diptera species for each urban site. (a) CAU-1, (b) CAU-2, (c) ZZP-1, (d) ZZP-2. Species are categorized based on a relative abundance threshold of 1.0% and a temporal duration threshold of 4 months: Core (abundance ≥ 1.0%, duration ≥ 4 months), Seasonal (abundance ≥ 1.0%, duration < 4 months), Background (abundance < 1.0%, duration ≥ 4 months), and Rare (abundance < 1.0%, duration < 4 months). Points and bars are colored by trophic guild: blue = detritivores, green = herbivores, red = predators/parasitoids/hematophagous. Dashed lines indicate classification thresholds. The five most abundant species in each category are labeled. Numeric labels on points indicate the number of overlapping species sharing identical coordinates.

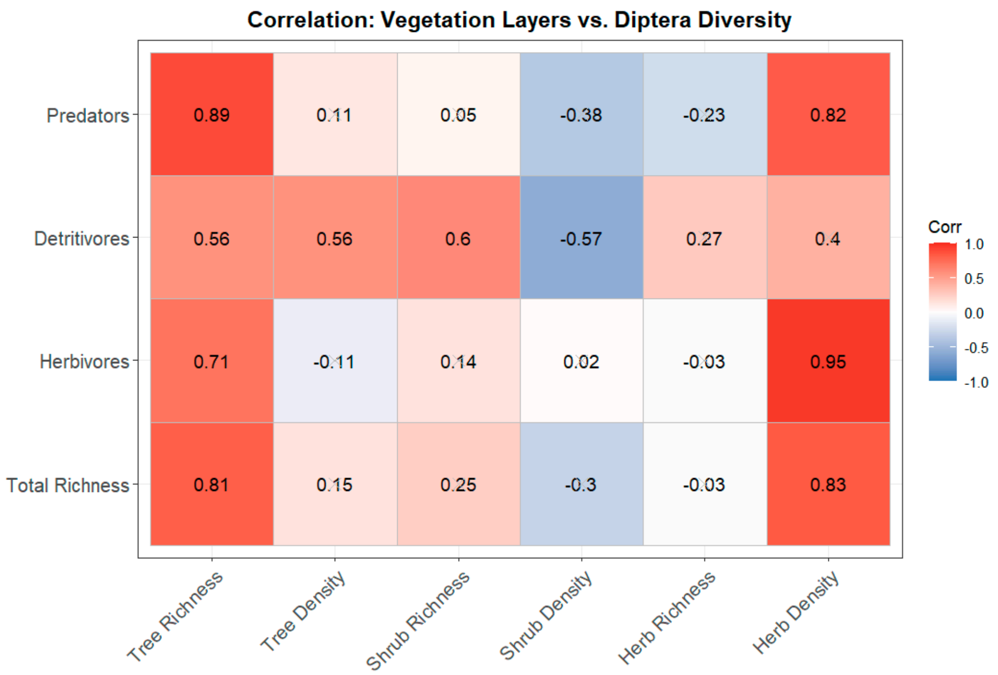

Figure S6. Correlation heatmap of vegetation structure and Diptera diversity. The heatmap displays the Pearson correlation strength (R) between stratified vegetation parameters (Tree, Shrub, and Herb layers) and major Diptera functional guilds. Red indicates positive correlation, while blue indicates negative correlation. Numbers within cells represent the correlation coefficients.

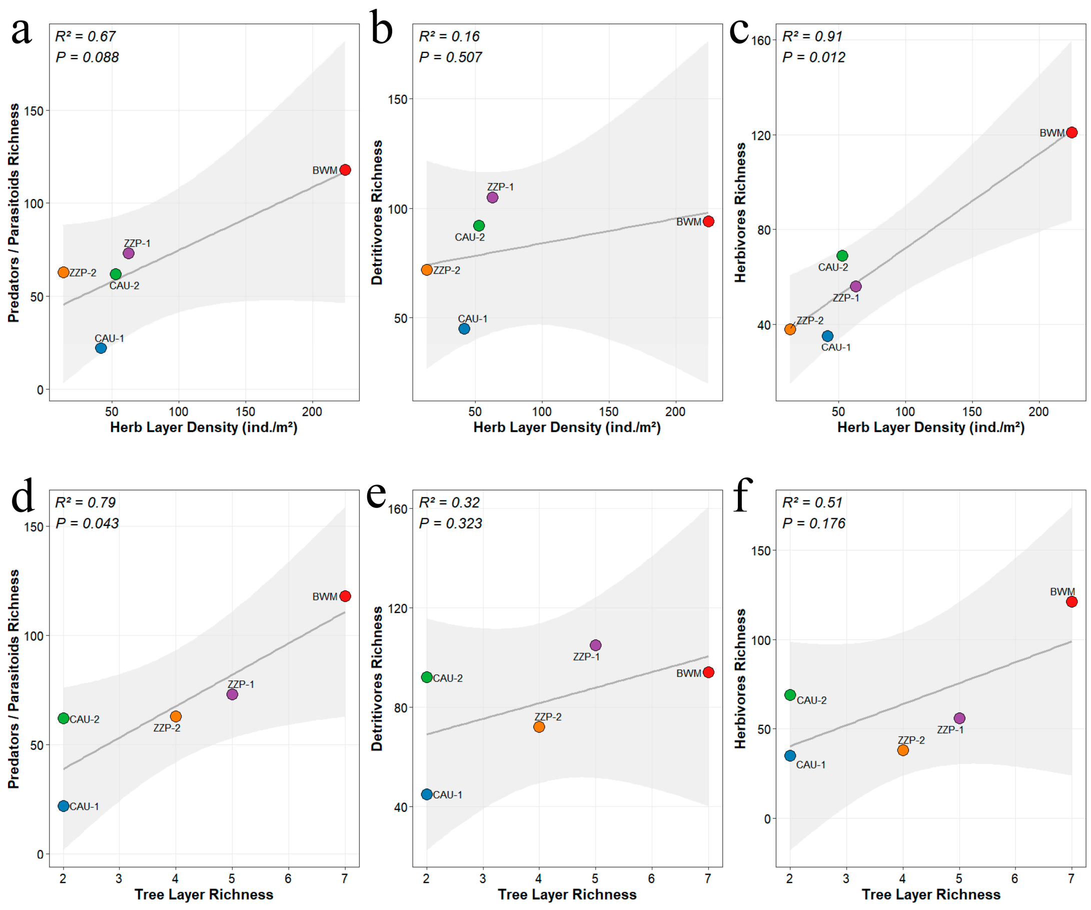

Figure S7. Impact of herb layer density and tree richness on Diptera functional guilds. Scatter plots show the relationships between key vegetation drivers and insect richness across five sampling sites. (a–c) The influence of herb layer density on (a) predators/parasitoids, (b) detritivores, and (c) herbivores. (d–f) The influence of tree layer richness on (d) predators/parasitoids, (e) detritivores, and (f) herbivores. Each plot includes a linear regression line (solid) with a 95% confidence interval (shaded). Individual sampling sites are color-coded.
